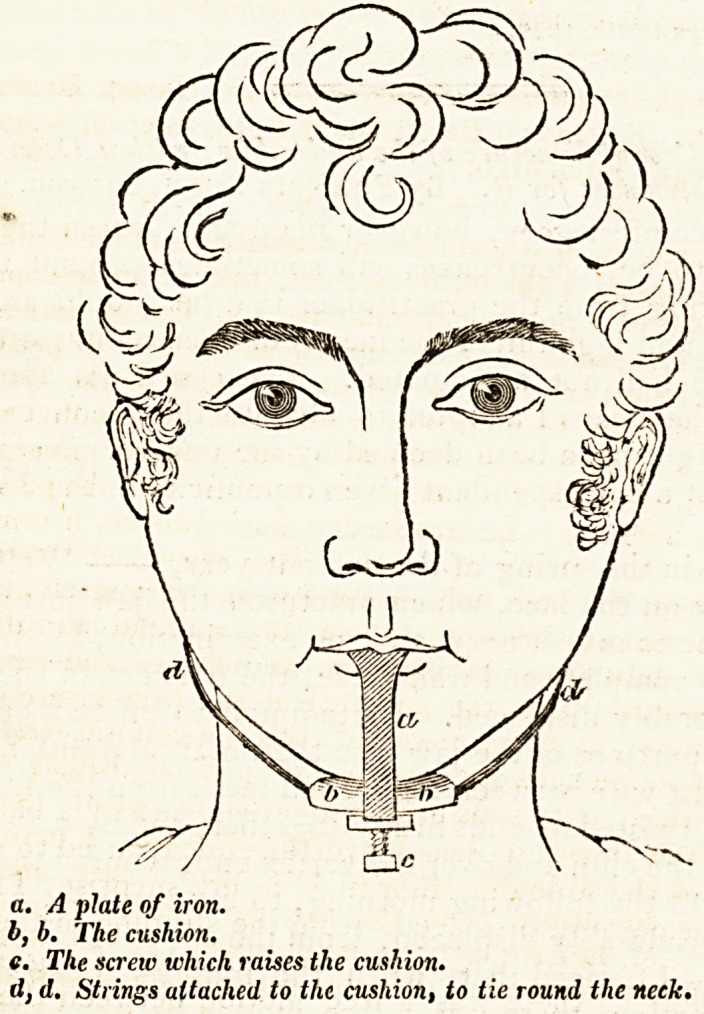# A Case of Fracture of the under Jaw; with a Description of an Instrument for It

**Published:** 1822-11

**Authors:** Francis Bush

**Affiliations:** Surgeon.


ART. VII.—*A Case of Fracture of the under Jaw; with a Description of an Instrument for it.* By FRANCIS BUSH, Surgeon.

IN the practice of surgery, however plain and beaten the path may appear to be, occurrences will sometimes present themselves, requiring from the practitioner that he should supply, by his talents and ingenuity, the means of overcoming difficulties which he had not anticipated. The case I am about to recite, with the means I adopted to obviate the inconvenience that arose, might have been deemed by me undeserving public notice, had not a correspondent given it publicity in the Journal for August.

Some time in the spring of the current year, —— Wolfe received a blow on the face, which fractured the jaw just at the chin: he came to my surgery, and, on examination, I found the fracture to be complete and transverse, the portions of the bone being considerably displaced. I attempted to effect a cure by bringing the portions of the jaw into their natural position, and having placed a wire between the second teeth from the fracture on each side, twisted its ends firmly together, and by a bandage placed under the chin and over the vertex endeavoured to retain the parts. On the following morning, to my surprise, I found they were considerably displaced, from the slipping of the wire on the teeth. I noticed that, when the muscles of deglutition were put in action, there was a free motion between the fractured parts. To confine the broken bone more effectually, I obtained an instrument, the principle of which will be best understood by examining the graphic sketch accompanying this communication. It is formed by a thin sheet of iron, an inch or more wide, bent so that one end of it may rest on the four front teeth, whilst the other passes under the chin: that part which rests on the teeth should be padded, and covered with leather or some soft substance, to prevent uneasiness; and between the chin and the iron a cushion is placed, which is raised or sunk by a screw, so as to give the exact pressure required. To the cushion a ribbon should be attached, to tie round the neck, to prevent the instrument from slipping off forward. This was fixed on the chin the day after the fracture had taken place, and the man was requested to call in the evening, if in pain. He did not call; but in the morning he came twice to my house, without seeing me, as I was engaged with an accouchement at some distance from home; and, in consequence of my absence, he applied to another surgeon, by whom the fracture was treated without the instrument I have described.

*a. A plate of iron.*

*b, b. The cushion.*

*c. The screw which raises the cushion.*

*d, d. Strings attached to the cushion, to tie round the neck.*

The only difference between this instrument and that fixed on Wolfe's chin, is that the latter was so bent as to suit his individual case; whereas the former is capable of accommodating itself to the chin of any person, from being lengthened or shortened at pleasure, by means of the screw under the cushion.

I called on Wolfe some weeks after, along with Mr. Mantell: we found the bone firmly united, but the ends as much displaced as when he first applied to me.

*Frome; September,* 1822.

## Figures and Tables

**Figure f1:**